# Rethinking neonatal *Escherichia coli* sepsis: the overlooked role of maternally transferred immunity

**DOI:** 10.3389/fimmu.2026.1849816

**Published:** 2026-06-02

**Authors:** Daolin Tang, Rui Kang

**Affiliations:** Department of Surgery, UT Southwestern Medical Center, Dallas, TX, United States

**Keywords:** escherichia coli, IgG, immunity, infant, sepsis

## Introduction

Neonatal sepsis remains a major cause of early-life morbidity and mortality, particularly among preterm infants and those requiring intensive care (1). Globally, neonatal sepsis and related neonatal infections are estimated to account for approximately 1.3 million incident cases and more than 550,000 newborn deaths annually, despite a sustained decline in age-standardized mortality rates over the past three decades (2, 3). Among Gram-negative pathogens, *Escherichia coli* continues to be one of the most frequent causes of both early- and late-onset bloodstream infection and is associated with considerable mortality as well as long-term neurodevelopmental sequelae (4, 5). Yet a fundamental question remains insufficiently addressed: why does invasive disease develop in only a minority of newborns despite the fact that exposure to *E. coli* begins almost immediately after birth?

**Figure 1:**
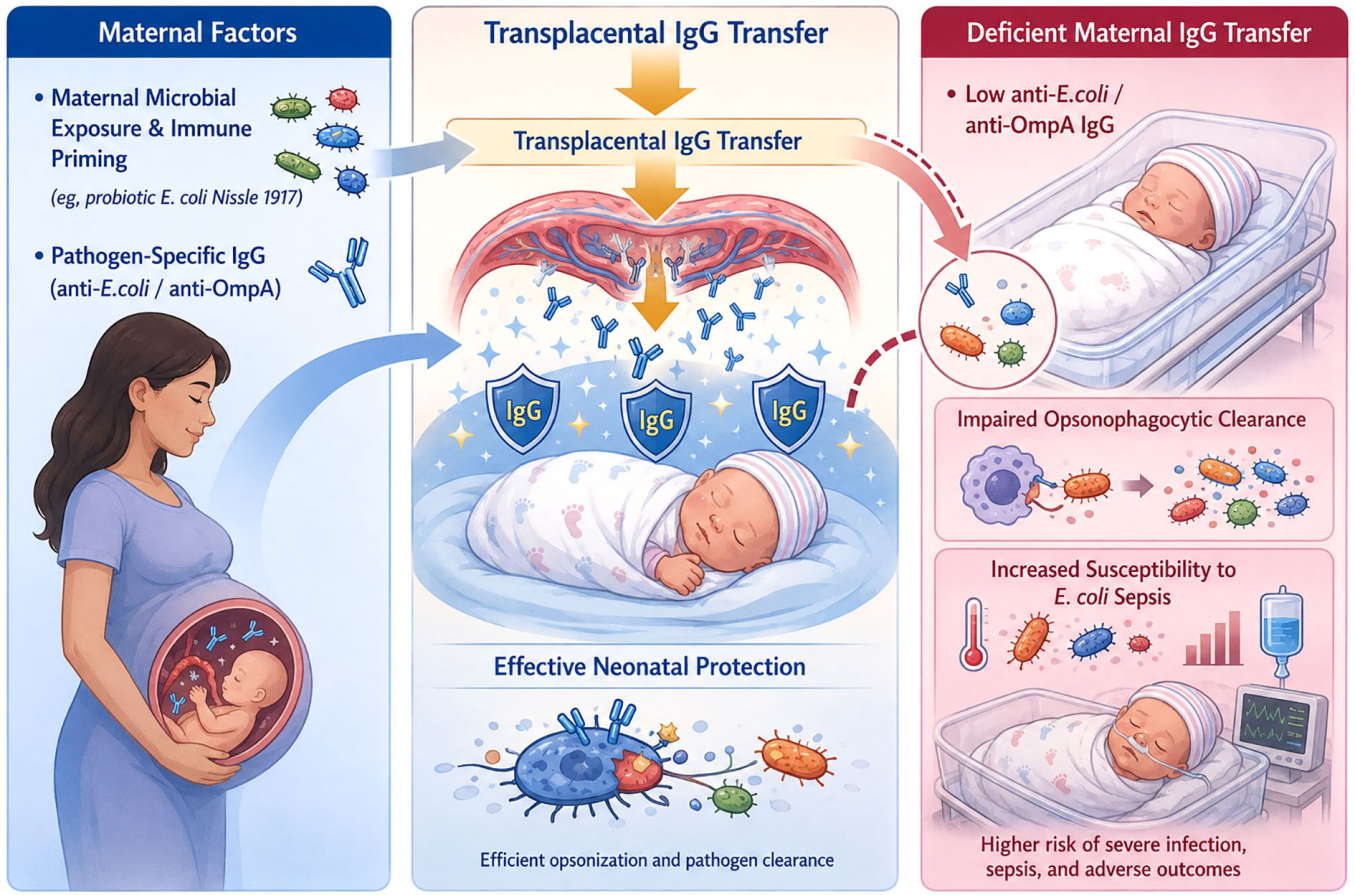
Proposed conceptual model of maternal immunity as a determinant of neonatal *Escherichia coli* sepsis susceptibility. Maternal microbial exposure and immune priming, including prior colonization by commensal or probiotic *E. coli* strains such as *E. coli* Nissle 1917, may promote the generation of pathogen-specific IgG antibodies, including anti-*E. coli* and anti-OmpA IgG. During late gestation, these antibodies are transferred transplacentally to the fetus, providing early postnatal protection through enhanced bacterial recognition and opsonophagocytic clearance. In contrast, deficient maternal IgG transfer may result in reduced pathogen-specific antibody levels, impaired bacterial clearance, and increased susceptibility to neonatal *E. coli* sepsis. The figure is conceptual and schematic; arrow thickness, spatial arrangement, and relative component size are not intended to represent quantitative effect sizes or experimentally measured transfer efficiencies.

The recent study by Diep and colleagues, published in *Nature*, provides an important new perspective on this question (6). Rather than focusing primarily on pathogen exposure or the intrinsic immaturity of neonatal immune responses, the authors direct attention to the immunological state in which the infant enters postnatal life. Their findings suggest that susceptibility to neonatal *E. coli* sepsis may depend less on exposure itself than on whether adequate maternally derived, pathogen-specific IgG, transferred transplacentally during late gestation, has reached the infant at the time of delivery (**Figure 1**). This perspective is conceptually important, as it shifts the discussion from a purely neonatal problem to one that also encompasses maternal immune status and vertical immune transfer through placental IgG transport. This transfer is mediated by the neonatal Fc receptor (FcRn), which actively transports maternal IgG across the placenta in a gestational age–dependent and subclass-selective manner (7, 8).

To better interpret these findings, it is important to briefly outline the study design. Methodologically, Diep et al. combined retrospective analysis of archived neonatal dried blood spot specimens with complementary preclinical mouse experiments. In the human cohort, dried blood spots collected on the first day of life for routine newborn screening were obtained from 100 infants who subsequently developed neonatal *E. coli* sepsis and compared with matched controls without infection. Controls were matched by sex, gestational age, and birth timing, thereby addressing several major variables known to influence maternally transferred immunity. Anti–*E. coli* IgG levels were quantified using endpoint antibody titers against pooled neonatal *E. coli* clinical isolates, including strains representing sequence types commonly associated with neonatal sepsis. Functional antibody activity was assessed using opsonophagocytic assays in human macrophage and neutrophil cell lines. This design allowed the authors to link not only antibody abundance, but also antibody-mediated antibacterial function, with subsequent neonatal sepsis susceptibility.

### Maternal antibody deficiency as a biologically plausible explanation for susceptibility

One of the most compelling observations in the study is that infants who subsequently developed *E. coli* sepsis had an approximately tenfold reduction in anti–*E. coli* IgG titers in dried blood spot samples collected within the first day of life. Anti-OmpA IgG titers were also reduced, and functional opsonophagocytic activity was diminished by roughly one order of magnitude in macrophage- and neutrophil-based assays (6). However, newborn blood spots from infants that developed sepsis compared with controls had similar overall IgG levels suggesting that susceptibility was not merely reflective of inadequate vertical transfer with diminished bulk antibodies among infected infants, but with a measurable reduction in both pathogen-specific IgG abundance and antibacterial effector function. The functional relevance of this finding is reinforced by the accompanying reduction in opsonophagocytic activity, reflecting impaired antibody-mediated bacterial tagging and subsequent phagocytic clearance, suggesting that difference do not simply levels of anti-*E. coli* IgG, and instead directly related to their anti-bacterial function.

This distinction is worth emphasizing. Neonatal susceptibility to infection has traditionally been attributed to developmental limitations in innate immunity, including reduced myeloid cell recruitment, active immune suppression, lower complement activity, and incomplete adaptive immune maturation (9–11). Although these factors are undoubtedly relevant, they do not fully explain why the vast majority of neonates, including many preterm infants, do not develop invasive disease despite near-universal bacterial exposure.

In this context, the findings by Diep et al. offer a more mechanistically satisfying explanation. Maternally transferred pathogen-specific antibodies may represent the principal protective layer that compensates for these developmental constraints during the first days and weeks of life. Seen from this perspective, the issue is not simply that the neonatal immune system is immature, but that in a subset of infants the maternal humoral buffer is insufficient.

This interpretation is consistent with the broader literature on maternal–fetal immune transfer, in which placental IgG has long been recognized as a major determinant of early-life protection (8, 12). What the present work contributes is disease-specific evidence that directly links deficiency in transferred antibodies to the risk of Gram-negative neonatal sepsis (6).

IgG subclass biology adds another important layer of complexity. In humans, placental transfer is not equivalent across subclasses, with IgG1 generally transferred most efficiently and IgG2 less efficiently (13). This is relevant because antibacterial responses, particularly those directed against bacterial surface polysaccharides and some bacterial antigens, often involve IgG2. Diep et al. found that the reduction in anti–*E. coli* antibodies was especially pronounced for IgG2, whereas total IgG recovery was not similarly reduced (6). This observation strengthens the argument that the defect was pathogen-specific, but it also highlights the important translational consideration that maternal vaccination strategies against *E. coli* may need to elicit antibody subclasses that are both functionally antibacterial and efficiently transferred across the placenta.

### Maternal microbial exposure as a determinant of neonatal immune protection

The murine preconception colonization experiments are, in our view, among the most thought-provoking aspects of the study. By showing that maternal colonization with *E. coli* Nissle 1917 (EcN) induces broadly cross-reactive IgG capable of protecting offspring, the authors introduce an important connection between maternal microbial ecology and neonatal infection resistance.

This observation aligns with an expanding body of work on the maternal microbiota–offspring immune axis. Previous studies have shown that maternal microbial signals influence fetal and neonatal immune development through transferred metabolites, cytokines, and antibodies (14, 15). However, the present study extends this concept by demonstrating direct protection against a clinically relevant invasive pathogen.

What makes this particularly interesting is that it encourages a broader interpretation of maternal colonization. Rather than considering maternal microbiota solely as a source of neonatal exposure, it may be more appropriate to regard it as part of an immunological priming system that shapes the antibody repertoire available to the newborn at birth.

This idea may also help explain some of the interindividual variation in neonatal sepsis risk that is not easily accounted for by gestational age, birth weight, or obstetric complications alone (16). Maternal immune history, shaped in part by prior microbial exposure and colonization patterns, may represent one of several underrecognized contributors to neonatal infection susceptibility (17).

### Clinical implications

From a translational standpoint, the study raises several clinically relevant possibilities.

One immediate implication concerns risk stratification. If low pathogen-specific maternal antibody levels are indeed associated with a substantially increased risk of neonatal sepsis, then serological assessment during late pregnancy or at delivery may deserve consideration, particularly in pregnancies already considered high risk. Existing obstetric practice already incorporates targeted screening for vertically relevant pathogens, and the current findings suggest that a similar logic could be extended to pathogen-specific humoral protection.

The study also has important implications for prevention. In particular, the identification of outer membrane protein A (OmpA)—a conserved surface protein located on the outer membrane of *E. coli* and exposed to host immune recognition (18)—as an immunologically relevant target is especially noteworthy. Because proteins on the bacterial surface are more readily accessible to circulating antibodies, OmpA represents a biologically plausible antigen through which maternally derived IgG may mediate neonatal protection. Although antigenic heterogeneity across invasive *E. coli* strains remains an important challenge, conserved outer membrane proteins such as OmpA may provide a rational foundation for the development of maternal immunization strategies. Given the established success of maternal vaccination against pathogens such as influenza virus and pertussis, these findings support further investigation into whether maternal vaccination strategies could be extended to neonatal Gram-negative sepsis.

The analogy to influenza virus and pertussis vaccination is mechanistically relevant because these vaccines protect neonates primarily by increasing pathogen-specific maternal IgG titers before delivery, followed by FcRn-mediated transplacental transfer to the fetus. In this sense, the Diep et al. findings provide a coherent biological rationale for considering maternal immunization against selected *E. coli* antigens: the goal would not be to stimulate neonatal immunity directly, but to increase the quantity and functional quality of protective maternal IgG available at birth. Whether conserved *E. coli* antigens such as OmpA can induce sufficiently broad and functional antibody responses remains an important question for vaccine development.

A further, albeit more exploratory, implication concerns microbiota-directed intervention. While still far from clinical application, the murine findings suggest that maternal microbial modulation before or during pregnancy could influence the quantity and quality of protective antibodies transferred to offspring.

### Remaining uncertainties

At the same time, several issues require caution. Most notably, the human component of the study remains retrospective. In the absence of paired maternal serum samples, placental transfer measurements, and maternal microbiome profiling, it is difficult to determine the precise origin of reduced neonatal antibody levels. Whether this reflects inadequate maternal immune priming, altered placental transfer efficiency, or other perinatal factors remains unresolved. This distinction is not merely academic, as it would directly influence the most rational preventive strategy. In addition, while OmpA is a promising target, the diversity of invasive *E. coli* strains means that broader antigenic validation will be necessary before any translational application can be realistically considered.

Gestational age deserves particular attention in interpreting these findings. Transplacental IgG transfer is most efficient during the third trimester, and preterm delivery may therefore reduce neonatal IgG levels independently of maternal antibody abundance. Thus, low anti–*E. coli* IgG in susceptible infants could reflect insufficient maternal antibody generation, interrupted placental transfer due to prematurity, impaired FcRn-mediated transport, or a combination of these mechanisms. Diep et al. partially addressed this confounder by matching sepsis cases and controls for gestational age and by showing that reductions in anti–*E. coli* IgG and opsonophagocytic activity persisted across gestational ages. Nevertheless, future prospective studies should include paired maternal and cord blood samples to distinguish low maternal antibody titers from inefficient or incomplete placental transfer. This distinction will be critical for determining whether the most rational intervention is maternal immunization, improved risk stratification of preterm infants, or postnatal antibody supplementation.

A further distinction concerns early-onset and late-onset neonatal sepsis. Maternally transferred IgG is biologically most intuitive as protection against early-onset disease, in which infection often reflects perinatal or intrapartum exposure. However, it may also influence late-onset disease by shaping host defense during the first weeks of life, when nosocomial exposure, intestinal colonization, and microbiome-related translocation become increasingly relevant. Diep et al. included infants with infection onset across the neonatal period, from the first day of life to several weeks after birth, and reported that reduced anti–*E. coli* IgG and opsonization activity were maintained regardless of postnatal age at infection onset. Even so, future studies should stratify early- and late-onset E. coli sepsis as biologically related but clinically distinct entities.

## Conclusion and outlook

The importance of this study lies not simply in the identification of an additional risk factor, but the way it changes the underlying framing of the problem. Rather than asking why neonates are inherently vulnerable to infection, it may be more productive to ask why most neonates remain protected despite constant exposure to potentially invasive organisms.

This shift in perspective is, in our opinion, one of the most valuable contributions of the work. It moves the field away from an exclusively deficit-based view of neonatal immunity and toward a model in which protection depends on the successful transfer of maternal immune memory.

Future studies should now focus on prospective validation of antibody thresholds, characterization of maternal microbiota–antibody relationships, and evaluation of targeted maternal immunization approaches.

If confirmed in larger clinical cohorts, these findings may have important implications for how neonatal Gram-negative sepsis is prevented—beginning not with the newborn, but with maternal immune preparedness.
